# O9-1 Physical activity change and stability patterns from adolescence to early adulthood: how activity domains and sedentary behaviour are associated with maintaining, increasing and decreasing activity?

**DOI:** 10.1093/eurpub/ckac094.065

**Published:** 2022-08-29

**Authors:** Tuula Aira, Tommi Vasankari, Olli Heinonen, Raija Korpelainen, Jari Parkkari, Kai Savonen, Arja Uusitalo, Maarit Valtonen, Jari Villberg, Sami Kokko

**Affiliations:** 1 Faculty of Sports and Health Sciences, University of Jyväskylä, Jyväskylä, Finland; 2 UKK Institute of Health Promotion Research, Tampere, Finland; 3 Paavo Nurmi centre & Unit for Health and Physical Activity, University of Turku, Turku, Finland; 4 Medical Research Center (MRC), University of Oulu and University Hospital of Oulu, Oulu, Finland; 5 Center for Life Course Health Research, University of Oulu, Oulu, Finland; 6 Tampere Research Center of Sports Medicine, Tampere, Finland; 7 Department of Clinical Physiology and Nuclear Medicine, Kuopio University Hospital, Kuopio, Finland; 8 Kuopio Research Institute of Exercise Medicine, Kuopio, Finland; 9 Clinic for Sports and Exercise Medicine, Foundation for Sports and Exercise Medicine, Helsinki, Finland; 10 Department of Sports and Exercise Medicine, Clinicum, University of Helsinki, Helsinki, Finland; 11 Research Center for Olympic Sports, Jyväskylä, Finland

**Keywords:** sports participation, youth, longitudinal study

## Abstract

**Background:**

Longitudinal studies demonstrate that physical activity (PA) declines on average from adolescence to early adulthood. However, some subgroups of adolescents increase activity while others decrease or maintain high or low activity. Determinants of change or maintenance of (in)activity may differ between subgroups and are valuable information for targeted health promotion. The purpose of this study was to identify PA patterns from adolescence to early adulthood, and also to explore how different activity domains and Sedentary Behaviour (SB) are associated with PA patterns.

**Methods:**

The data of this observational cohort study (collected in 2013/2014 and 2017/2018) consisted of 254 Finns at age 15 and 19 participating the Health Promoting Sports Club study. K-means cluster analysis for longitudinal data was performed to identify participant clusters (patterns) based on their accelerometry-measured moderate-to-vigorous PA (MVPA). Associations of sports club participation (SC), active commuting (AC), and SB with PA patterns were examined by logistic regression analysis.

**Results (preliminary):**

Five MVPA patterns were identified: inactivity maintainers (n = 71), activity maintainers (n = 70), decreasers from moderate (to low) PA (n = 61), decreasers from high (to moderate) PA (n = 32), and increasers (n = 20). At age 15, SC participation (41-97%) and AC (47-75%) were common in all the patterns. By age 19, clear dropout from these activities was prevalent (SC participation mean 32%, AC 31-63%). Maintained SC participation was associated with a higher likelihood of belonging to the decreasers from high PA (OR = 11.2, CI = 1.4-90.0) and to the combined group of increasers and activity maintainers (OR = 3.6, CI = 1.8-7.4); also with a lower likelihood of being an inactivity maintainer (OR = 0.1, CI = 0.02-0.2). Dropout from SC was related to a higher likelihood of being a decreaser from high PA (OR = 10.9, CI = 1.3-90.7). Maintenance/adoption of AC was associated with a lower likelihood of being an inactivity maintainer (OR = 0.3, CI = 0.1-0.7). Decreased SB was related to a higher likelihood of belonging to the activity maintainers and increasers (OR = 0.96, CI = 0.93-0.98).

**Conclusions:**

PA patterns diverge greatly over the transition to adulthood. Changes in SC participation, AC, and SB show different associations with diverging PA patterns. Hence, tailored PA promotion is recommended.

